# Streamlined protein expression and purification using cleavable self-aggregating tags

**DOI:** 10.1186/1475-2859-10-42

**Published:** 2011-06-02

**Authors:** Lei Xing, Wei Wu, Bihong Zhou, Zhanglin Lin

**Affiliations:** 1 Department of Chemical Engineering, Tsinghua University, One Tsinghua Garden Road, Beijing 100084, China

## Abstract

**Background:**

Recombinant protein expression and purification remains a fundamental issue for biotechnology. Recently we found that two short self-assembling amphipathic peptides 18A (EWLKAFYEKVLEKLKELF) and ELK16 (LELELKLKLELELKLK) can induce the formation of active protein aggregates in *Escherichia coli *(*E. coli*), in which the target proteins retain high enzymatic activities. Here we further explore this finding to develop a novel, facile, matrix-free protein expression and purification approach.

**Results:**

In this paper, we describe a streamlined protein expression and purification approach by using cleavable self-aggregating tags comprising of one amphipathic peptide (18A or ELK16) and an intein molecule. In such a scheme, a target protein is first expressed as active protein aggregate, separated by simple centrifugation, and then released into solution by intein-mediated cleavage. Three target proteins including lipase A, amadoriase II and β-xylosidase were used to demonstrate the feasibility of this approach. All the target proteins released after cleavage were highly active and pure (over 90% in the case of intein-ELK16 fusions). The yields were in the range of 1.6-10.4 μg/mg wet cell pellet at small laboratory scale, which is comparable with the typical yields from the classical his-tag purification, the IMPACT-CN system (New England Biolabs, Beverly, MA), and the ELP tag purification scheme.

**Conclusions:**

This tested single step purification is capable of producing proteins with high quantity and purity. It can greatly reduce the cost and time, and thus provides application potentials for both industrial scale up and laboratorial usage.

## Background

**Figure 1:**
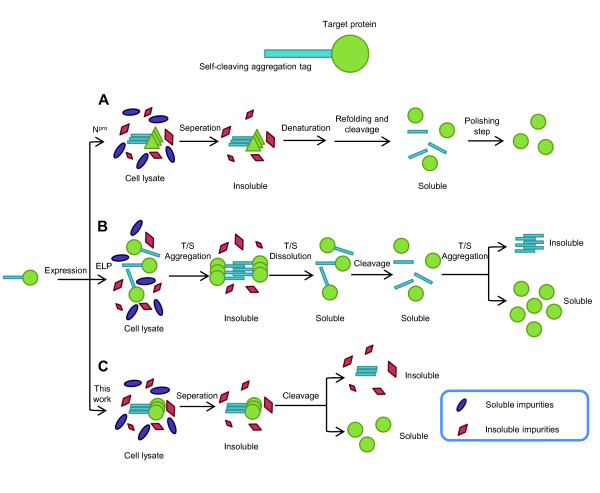
**Strategies of quick protein expression and purification using self-cleaving aggregation tags**. (A) N^pro ^fusion technology, fusions expressed as inclusion bodies are separated, refolded, and cleaved, followed by a polishing step to remove soluble N^pro^. (B) The ELP system and (C) this work, the self-cleaving aggregation tags are based on the combination of a self-cleavable intein and a self-aggregating peptide (ELP) or a self-assembling peptide (18A or ELK16). T/S: temperature or salt-mediated.

For recombinant protein expression and production, the use of affinity tags such as polyhistidine (his-tag), glutathione transferase (GST), and the self-cleavable inteins have greatly reduced chromatography steps and increased yields [1,2]. In recent years, two further simplified protein expression and purification schemes have emerged, both of which take advantage of induced protein aggregates that can be easily recovered by centrifugation, followed by tag cleavage (Figure 1). The first scheme uses a self-cleaving aggregation tag, N^pro^, an autoprotease from the swine fever virus (CSFV) [3]. However, this approach yields the classical inactive inclusion bodies and demands an additional and sometimes tedious step of refolding, and may require a polishing step to eliminate the residual N^pro ^fragments (Figure 1A). The second scheme utilizes an elastin-like peptide (ELP) in combination with a self-cleavable intein. The ELP tag facilitates purification by cycles of aggregation and solubilization of the target protein mediated by temperature and/or salt shifts [4,5], which is then released into solution by intein-mediated cleavage between the tag and the target protein (Figure 1B). The disadvantage of this approach is that it requires several manipulation steps, or "inverse transition cycling," modulated by temperature or salinity, which requires optimization for different proteins and ELP.

In previous studies, we found that two short terminal self-assembling peptides, an amphipathic alpha peptide 18A (EWLKAFYEKVLEKLKELF) (Wu W, Xing L, Zhou B, Cai Z, Chen B, Lin Z: Assembly of active protein aggregates in vivo induced by terminally attached amphipathic peptide, submitted) and a beta peptide ELK16 (LELELKLKLELELKLK) [6] can induce the formation of highly active enzyme aggregates *in vivo *[7-10]. This has inspired us to devise a protein expression and purification scheme in combination with a self-cleavable intein. The advantageous distinction between these two peptide tags and the ELP tag is that for the 18A and ELK16 peptides, the active protein aggregates are obtained during protein expression, and thus only one single step is required for intein-mediated tag cleavage to release the active target proteins (Figure 1C). Moreover, peptides 18A and ELK16 (18 aa and 16 aa in length, respectively) are much smaller in size compared with ELP tags (450-550 aa). Here we use three target proteins, *Bacillus subtilis *lipase A (LipA) [11], *Bacillus pumilus *xylosidase (XynB) [12,13], *Aspergillus fumigatus *amadoriase II (AMA) [14] to demonstrate the feasibility of this streamlined matrix-free protein expression and purification approach.

## Methods

### Materials

The restriction enzymes *Nde*I, *Hind*III, *EcoR*I, *Dpn*I, *Spe*I and the T4 DNA ligase were from either New England Biolabs (Beverly, MA) or Takara (Dalian, China). The pfu DNA polymerase was from Tiangen (Beijing, China). The vector pTWIN1 was from New England Biolabs. The vector pET30a (+) and strain *E. coli *BL21 (DE3) were from Novagen (Madison, WI). The kits for DNA purification, gel extraction, and plasmid mini-prep were from Tiangen (Beijing, China). The oligonucleotides used for gene synthesis and amplification were synthesized by Invitrogen (Carlsbad, CA), and the sequencing was performed by Sunbiotech (Beijing, China). 4-nitrophenyl palmitate (*p*NPP) and 4-nitrophenyl β-D-xyloside (*p*NPX) for enzyme assays were obtained from Sigma-Aldrich (St. Louis, MO). Horseradish peroxidase and 4-aminoantipyrine were from Sangon (Shanghai, China). All other chemicals were of analytic grade.

### Plasmid construction

**Figure 2:**
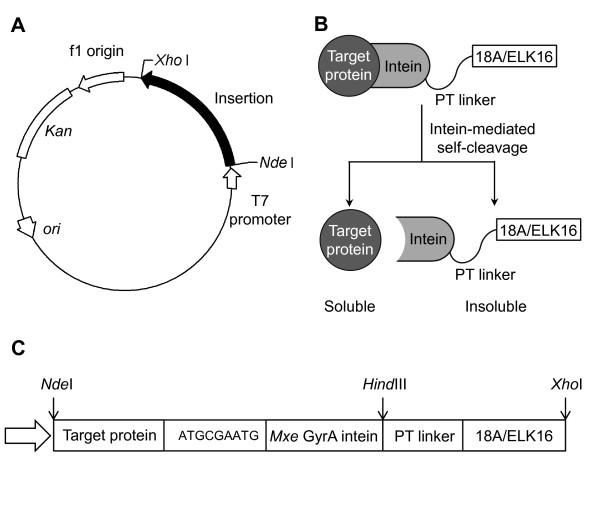
**Construction for fusion proteins (target protein-intein-18A/ELK16)**. (A) Plasmid map. (B) Schematic of fusion constructs. (C) Schematic of inserted fusion sequences. The short segment ATGCGAATG encoding MRM was inserted between the genes encoding target protein and *Mxe *GyrA intein to facilitate the intein-mediated cleavage.

Plasmids encoding the fusion proteins (Figure 2) for the target proteins with 18A peptide (18A) or ELK16 peptide (ELK16) attached were based on the plasmids pAc18A (Wu W, Xing L, Zhou B, Cai Z, Chen B, Lin Z: Assembly of active protein aggregates in vivo induced by terminally attached amphipathic peptide, submitted) and pET30a-LipA-ELK16 [6], constructed previously in our lab. The gene encoding lipase A was first amplified, and overlapped with the *Mxe *GyrA intein sequence amplified from pTWIN1 plasmid [15], using pfu DNA polymerase and primers listed in Additional file 1, Table S1. The fusion gene was then digested with *Nde*I and *Hind*III, and then inserted into the similarly digested vector pAc18A or pET30a-LipA-ELK16, yielding pET30a-LipA-I-18A, and pET30a-LipA-I-ELK16. Plasmids encoding other fusion proteins were similarly constructed using primers in Additional file 1, Table S1. *E. coli *BL21 (DE3) cells were used throughout for cloning and fusion protein expression. A second intein, the *Ssp *DnaB intein [16], which can be cleaved by pH and temperature shifts at its C-terminus, was also tested in a similar construction (fused to the N-terminus of a target protein). However, the cleavage efficiency was less desirable and thus this intein was not further pursued.

### Expression

*E. coli *BL21(DE3) cells harbouring pET30a-LipA/XynB/AMA-I-18A or pET30a-LipA/XynB/AMA-I-ELK16 were inoculated into Luria-Bertani (LB) medium supplemented with 50 mg/l kanamycin and incubated at 37°C with shaking (250 rpm). Isopropyl β-D-1-thiogalactopyranoside (IPTG) was added to a final concentration of 0.2 mM to initiate protein expression when cell optical density (OD_600_) reached 0.4-0.6. The cultures were then continued for an additional 6 h at 30°C, and then harvested by centrifugation at 8,000 rpm for 20 min and pellets were stored at-70°C for further assay and analysis.

### Protein purification by intein-mediated cleavage and SDS-PAGE analysis

Harvested cell pellets were re-suspended in buffer B1 (20 mM Tris-HCL,500 mM NaCl, 1 mM disodium edetate (EDTA), pH 8.5) to 10 OD_600 _culture/ml, followed by sonication (Ultrasonic crasher, Scientz JY92-IIN, Ningbo, China). One OD_600 _cell culture was determined to be 1.38 ± 0.17 mg wet cell pellets. The lysates were then separated by centrifugation at 11,000 rpm for 10 min at 4°C and the soluble fractions were collected. The precipitates were washed twice with buffer B1, resuspended in a same volume of Buffer B3 (20 mM Tris-HCl, 500 mM NaCl, 1 mM EDTA and 40 mM dithiothreitol (DTT), pH 8.5).

Different cleavage conditions (4°C/25°C and 3 h/24 h, all at pH 8.5) were tested. At 25°C, incubation of the samples for 3 h and 24 h generated similar amount of soluble lipase A in the supernatant (data not shown). At 4°C, it was found that incubation of the sample for 24 h yielded a higher amount of lipase A in the supernatant than 3 h (data not shown). Two other different pH values (5.6, 7.0) were also tested and found to yield similar results. Since low temperature is favourable for protein stability, we performed all subsequently cleavage reactions at 4°C for 24 h.

The amounts of proteins in all samples were determined densitometrically with Quantity One software (Bio-Rad Laboratories, Hercules, CA) by denaturing polyacrylamide gel electrophoresis (SDS PAGE, 12%) followed by staining with Coomassie Brilliant Blue G-250, using bovine serum albumin (BSA) as standard.

The nucleic acid contamination was determined by the ratio of absorbance at 260 nm and 280 nm of the released target proteins, using a nucleic acid and protein analyser DU^® ^640 (Beckman Coulter, Brea, CA).

### Activity assay

The activities of target proteins were measured in 96-well micro plates with a SPECTRAMAX M2 microtiter reader (Molecular Device, Sunnyvale, CA), following standard procedures [17-19]. In detail, the β-xylosidase and lipase A assays were carried out at 37°C by monitoring the formation of *p*-nitrophenol (*p*NP) following A_405 _(ε, 18.7 cm^2^/μmol). The substrate *p*NPP was first dissolved in 2-propanol, and then mixed with reaction buffer (50 mM sodium phosphate buffer, pH 8.0, together with 1 mg/ml Arabic gum and 2.07 mg/ml sodium deoxycholate) by vortexing, resulting in final concentration of *p*NPP 1.5 mg/ml. The β-xylosidase reaction was performed in 180 μl of reaction system (50 mM phosphate buffer, pH 6.0, 2.5 mM *p*-nitrophenyl β-D-xylopyranoside). The amadoriase activity was measured at 37°C by monitoring the formation of a quinone dye following A_555 _(ε, 39.2 cm^2^/μmol) in a peroxidase-coupling reaction. The reaction mixture contained 100 mM potassium phosphate buffer (pH 8.0), 2.7 purpurogallin units of peroxidase, 0.45 mM 4-aminoantipyrine, 0.5 mM *N*-ethyl-*N*-(2-hydroxy-3-sulfopropyl)-*m*-toluidine (TOOS), and 5.0 mM D-fructosyl-glycine in a total volume of 180 μl. The reactions all started after mixing 175 μl of substrate solution and 5 μl of enzyme. One unit of enzyme activity was defined as the amount of enzyme that produced one μmol H_2_O_2 _or *p*-nitrophenol per min.

## Results

### Fusion construction

The general scheme for fusion construction is shown in Figure 2. A commonly used intein, the *Mxe *GyrA intein [15] was incorporated. This intein can be self-cleaved at its N-terminus by the addition of a thiol agent such as dithiothreitol (DTT), and was fused to the C-terminus of a target protein, with three additional amino acid residues (MRM) added to facilitate cleavage [5]. The self-assembling peptide, 18A or ELK16, was fused downstream to the *Mxe *GyrA intein via a PT type linker [20]. A second intein, the *Ssp *DnaB intein [16], which can be cleaved by pH and temperature shifts at its C-terminus, was also tested in a similar construction (fused to the N-terminus of the target protein). However, the cleavage efficiency was less desirable and thus it was not further pursued.

### Protein aggregation and self-cleavage for peptide 18A and ELK16 fusions with target proteins

**Figure 3:**
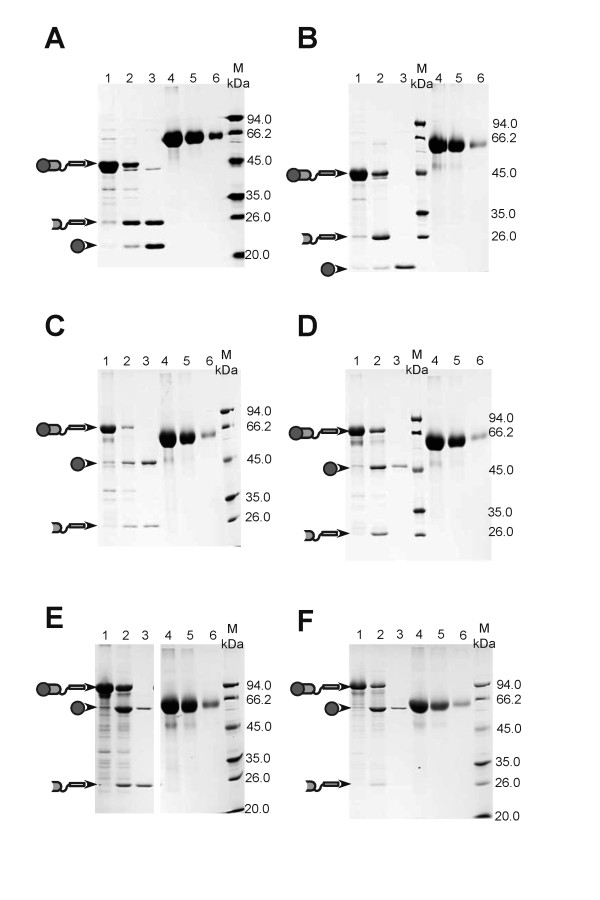
**Fusion protein (target protein-intein-18A/ELK16) expression and cleavage**. (A) LipA-I-18A. (B) LipA-I-ELK16. (C) AMA-I-18A. (D) AMA-I-ELK16. (E) XynB-I-18A. (F) XynB-I-ELK16. For (A-F): lane 1, insoluble fraction of cell lysate; lane 2, insoluble fraction of cleaved fusion protein; lane3, soluble fraction of cleaved fusion protein; lane 4, 5 and 6, bovine serum albumin (BSA) standards, at 6, 3 and 0.75 μg/lane, respectively.

Fusions for lipase A were first tested. The fusion LipA-*Mxe *GyrA intein-18A (LipA-I-18A) was successfully expressed largely as insoluble aggregate (lane 1 in Figure 3A), at a level estimated at 34.1 μg/mg wet cell pellet (Table 1, calculated from three independent clones). The aggregate was separated and subjected to cleavage with 40 mM DTT at 4°C for 24 h to release lipase A, and then the insoluble and soluble fractions were analyzed. About 78% of the LipA-I-18A aggregate was cleaved (Table 1, the cleavage efficiency is defined as the mass ratio of the cleaved aggregate over the total aggregate), and 10.4 μg/mg wet cell pellet free lipase A was obtained in the soluble fraction, which accounted for 87% of lipase A that could be released from the cleaved LipA-I-18A aggregate, thus the remaining 13% was likely entrapped in the insoluble fraction after cleavage. The mass percent recovery (defined as the mass ratio of the free protein released into the soluble solution after cleavage over the total free protein that could be theoretically obtained from the respective protein aggregate, assuming a complete cleavage and release) was thus 68%. The released lipase A was highly active, estimated at 72.8 units/mg, comparable with that of the conventionally purified lipase A, which was about 120 units/mg [21].

**Table 1:** Protein quantification and activity assays^a^

Product protein (molecular weight)	Aggregates^b ^(μg/mg wet cell pellet)	Quantity of purified protein^b ^(μg/mg wet cell pellet)	Specific activity (units/mg)	Specific activity reported in the literature (units/mg)	Cleavage efficiency^c^	Percent recovery (mass)^d^
From 18A fusions						
LipA (21 kDa)	34.1 ± 4.1	10.4 ± 0.3	72.8 ± 3.9		(78 ± 2)%	(68 ± 7)%
AMA (49 kDa)	19.1 ± 1.4	7.9 ± 1.0	2.3 ± 0.5		(80 ± 1)%	(62 ± 3)%
XynB (61 kDa)	18.1 ± 2.0	1.6 ± 0.2	2.6 ± 0.3		(69 ± 3)%	(14 ± 1)%
From ELK16 fusions						
LipA	31.0 ± 4.1	8.3 ± 0.7	133.4 ± 2.0	120 (ref. [21])	(73 ± 1)%	(59 ± 4)%
AMA	23.2 ± 2.9	4.0 ± 0.1	1.8 ± 0.3	1.9-2.5 (ref. [14])	(65 ± 2)%	(27 ± 3)%
XynB	17.6 ± 0.5	2.9 ± 0.1	1.5 ± 0.1	0.072-25.2^e ^(ref. [13])	(57 ± 2)%	(23 ± 1)%

^a^The experiments were carried out in triplicate with three independent expression clones. ^b^Yield of protein from LB culture with wet cell weight of 2.66 ± 0.99 mg/ml. ^c^Cleavage efficiency was calculated by dividing the amount of cleaved protein aggregate over that of the total aggregate before cleavage. ^d^Percent recovery in terms of mass was calculated by dividing the mass of the free protein released into the soluble solution after cleavage over the mass of the total free protein that could be theoretically obtained from the respective protein aggregate, assuming a complete cleavage and release. ^e^Specific activity of xylosidase obtained from two repeated steps of purification with a DEAE-Sepharose CL-6B or DEAE-Sephadex A-50 column was 2.4 units/mg and 1.98 units/mg, respectively.

However, although most intracellular protein impurities had been removed (lane 1 in Figure 3A), there was one peculiar band attributable to the intein-18A fragment (I-18A) appearing in the soluble fraction after DTT cleavage. This suggests that I-18A cleaved from LipA-I-18A remained partially soluble, perhaps because intein itself is difficult to be driven into aggregation by the peptide 18A. However, it should be noted that if I-18A does not interfere downstream applications, this expression and purification scheme should be satisfactory, as the combined amount of free lipase A and I-18A was greater than 90% of the total soluble protein (lane 3 in Figure 3A), based on the densitometry analysis using Quantity One software (Bio-Rad Laboratories). For the self-assembling peptide ELK16, the fusion LipA-*Mxe *GyrA intein-ELK16 (LipA-I-ELK16) produced a much more favourable result. As can be seen from Figure 3B, there is only one significant band corresponding to lipase A in the soluble fraction, which was about 92% pure as estimated densitometrically (lane 3 in Figure 3B). In this case, the intein-ELK16 fragment (I-ELK16) was largely insoluble. The expression level for LipA-I-ELK16 aggregate was estimated at 31.0 μg/mg wet cell pellet, and the cleavage efficiency for LipA-I-ELK16 and the mass percent recovery for lipase A were about 73% and 59%, respectively, and about 8.3 μg/mg wet cell of free lipase A was obtained in the soluble fraction. Again, the specific activity for lipase A released from the LipA-I-ELK16, at 133.4 units/mg, was comparable with that reported in the literature, and slightly higher than that released from the LipA-I-18A fusion. It is also interesting to note that lipase A (LipA) in either LipA-ELK16 aggregate [6] or LipA-intein-ELK16 (LipA-I-ELK16) aggregate (this study, data not shown) showed little hydrolytic activity against the substrate *p*NPP, but once it was released from the LipA-I-ELK16, it was highly active. Along this line, it should be added that lipase A, being a unique lid-less lipase [11], has a more exposed hydrophobic active site, and thus its specific activity might be more sensitive to its microenvironment, and to expression and purification conditions.

Similar aggregation and cleavage results were obtained for amadoriase II (AMA) and xylosidase (XynB), with specific activities comparable to those prepared by traditional expression and purification methods (Table 1 and the references therein, and Figure 3C-F). More specifically, for the amadoriase fusions, AMA-Mxe GyrA intein-18A (AMA-I-18A) and AMA-Mxe GyrA intein-ELK16 (AMA-I-ELK16), active aggregates at levels of about 19.1 μg/mg wet cell pellet and 23.2 μg/mg wet cell pellet, respectively, were obtained, respectively, which were cleaved at an efficiency of 80% and 65%, respectively. For AMA-I-18A fusion (Figure 3C), the free amadoriase released into solution was estimated at 7.9 μg/mg wet cell pellet, or 62% of the theoretic amount of amadoriase contained in the total AMA-I-18A aggregate. Interestingly, for AMA-I-ELK16 fusion (Figure 3D), the free amadoriase released into solution was lower, at 4.0 μg/mg wet cell pellet, or about 27% of the amadoriase contained in the total AMA-I-ELK16 aggregate. Thus, the majority of the cleaved amadoriase very likely remained to be entrapped in the ELK16 aggregate.

For the xylosidase fusions (Figure 3E, F), the expression of XynB-Mxe GyrA intein-18A (XynB-I-18A) and XynB-Mxe GyrA intein-ELK16 (XynB-I-ELK16) yielded active aggregates at levels of 18.1 μg/mg wet cell pellet and 17.6 μg/mg wet cell pellet, respectively. However, a much lower amount of free xylosidase was released into the soluble fraction after cleavage for both fusions, at 0.9 μg/mg wet cell pellet and 1.5 μg/mg wet cell pellet, respectively. Again, the majority of the cleaved xylosidase very likely remained to be entrapped in both the 18A and ELK16 aggregates, and the fact that xylosidase itself is prone to aggregation might exacerbate the entrapment. For example, we have observed that when expressed alone in *E. coli*, it often accumulates in inclusion bodies.

Additionally, for all the six fusions (with the 18A or ELK16 tag), the ratios of absorbance at 260 nm and 280 nm (A_260_/A_280_) for the released free target proteins were determined to be 0.75 ± 0.09, suggesting the samples contained a small amount of nucleic acid contamination, estimated at about 1.5% [22], but this is comparable to that for those proteins purified using other quick methods such as the his-tag approach in our lab (with A_260_/A_280 _around 0.73).

## Discussion

In this work, we report a streamlined protein expression and purification approach for *E. coli *using two self-cleaving aggregation tags, which comprise of a self-assembling amphipathic peptides 18A or ELK16, and a self-cleavable intein molecule. The peptide drives the target protein into active aggregate that can be easily recovered by centrifugation, and thus greatly simplifies the separation, while at a later point the intein molecule mediates the cleavage and release of the target protein from the aggregate and into solution.

Comparing these two self-cleaving aggregation tags, I-ELK16 performs better than I-18A, since for the latter, the I-18A fragment cleaved from the target protein-I-18A is partially soluble and contaminates the target protein (lane 3 in Figure 3A, C, E), suggesting that I-18A aggregate is less stable than I-ELK16. However, if I-18A does not interfere downstream applications, then it should be satisfactory as the protein impurities other than the target protein and the I-18A fragment often accounted for less 10% of the total soluble proteins (lane 3 in Figure 3A, C, E). For both of these two aggregation tags, the specific activities of released proteins were rather similar, which suggested that the tags did not interfere with the correct folding of the target protein, similar to the ELP aggregation tags [5]. In addition, three different pH values (5.6, 7.0 and 8.5) were tested for intein-mediated cleavage, which led to similar results (data not shown). This offers the possibility of tag cleavage at a range of pH values from 5.6 to 8.5. Lastly, we also wish to point out that these 18A or ELK16 tags do not impair growth of the host *E. coli *cells at 30°C, which is advantageous than the ELP tags where seemingly more stringent growth conditions are required [4].

The yields for released and highly pure proteins in our work are in the range of 1.6-10.4 μg/mg wet cell pellet (Table 1) at small laboratory scale. It has been reported that the average yield of the ELP tag purification scheme is 15 μg/mg dry cell weight [1,4], or 5 μg/mg wet cell pellet, since the dry cell weight is about one third to the wet cell weight for *E. coli *[9]. Therefore, the yield from our approach is comparable to that of the ELP tag purification scheme, and thus higher than those of the IMPACT-CN system (New England Biolabs, Beverly, MA), and the classical his-tag purification [1]. The approach we describe here however eliminates the usage of costly affinity resin and protease (if applicable), or cycles of precipitation and solubilization. Our approach thus should have potentials for both industrial scale up and laboratorial usage. It has been estimated that the total purification cost of the ELP tag purification scheme is about 10% of the next simplest his-tag approach [1]. Since our approach is even simpler (Figure 1), the total cost might be further reduced or is at least comparable to that of the ELP tag purification scheme. However, it should be noted that the use of DTT in our work for fusion cleavage might limit the spectrum of proteins that could be purified, since DTT can disrupt the disulfide bond in a protein. In this case, a cleavable site that does not require DTT would be preferable.

## Conclusions

In conclusion, the streamlined protein expression and purification approach we described here can greatly reduce the use of affinity resins, and the cost and time, and is capable of producing proteins with reasonable quantity and purity (over 90% in the case of intein-ELK16 fusions), which can be further processed if higher purity is desired. We surmise that this approach is also particularly suitable for producing enzymes for high-throughput studies, where both simplicity and economy are critical enabling factors [23].

## Competing interests

The authors declare that they have no competing interests.

## Authors' contributions

LX designed part of the experiments, performed most of the experiments, and prepared the manuscript draft. WW designed and performed part of the experiments. BZ participated in the enzymatic assays and instrumental analyses. ZL conceived the study, designed and supervised the experiments, and revised the manuscript. All authors read and approved the final manuscript.

## Supplementary Material

Additional file 1 **Table S1**. The primers used in this work. Click here for file
