# Supplementary material for: Streamlined protein expression and purification using cleavable self-aggregating tags
Source: Microb Cell Fact. 2011 Jun 2;10:42. doi: 10.1186/1475-2859-10-42 (PMC3124420; doi:10.1186/1475-2859-10-42)
Supplement: Additional file 1 — Table S1. The primers used in this work. [file 1475-2859-10-42-S1.DOC]

Table S1 Oligos used in this work

| Primer name | Nucleotide sequence^1^ | Description |
| --- | --- | --- |
| M-LipA-For | 5’-GCGATACATATGCACCATCACCATCA-3' (*Nde*I) | Used for amplification of LipA and *Mxe* GyrA intein sequence for construction of pET30a-LipA-I-18A and pET30a-LipA-I-ELK |
| M-OVLP-Rev | 5’-GCATCTCCCGTGATGCACATTCGCATATTCGTATTCTGGCCCC-3’ |
| M-OVLP-For | 5’-GGGGCCAGAATACGAATATGCGAATGTGCATCACGGGAGAT-3’ |
| M-LipA-Rev | 5’-ATTTTAAAGCTTAGCGTGGCTGACGAACCCGTTC-3’ (*Hind*III) |
| XynB-NdeI-For | 5’-ATGAGCACATATGAAGATTATCAATCCAGTGCTC-3’ (*Nde*I) | Used for amplification of XynB for construction of pET30a-XynB-I-18A and pET30a-XynB-I-ELK |
| XynB-SpeI-Rev | 5’-CGAGCAACTAGTGCATCTCCCGTGATGCACATTCGCATTTCGTCTGTTTCCTCATAAC-3’ (*Spe*I) |
| AMA-NdeI-For | 5’- TTCTGGACATATGGCGGTAACCAAGTCATC-3’ (*Nde*I) | Used for amplification of AMA for construction of pET30a-AMA-I-18A and pET30a-AMA-I-ELK |
| AMA-SpeI-Rev | 5’-GGTGGTACTAGTGCATCTCCCGTGATGCACATTCGCATTAACTTGGAAATATCTCTATA-3’ (*Spe*I) |

^1^The underlined nucleotides indicate restriction sites.
